# Cancer predisposition syndromes: an imaging review

**DOI:** 10.1186/s40644-026-01003-1

**Published:** 2026-02-17

**Authors:** Livja Mertiri, Huy Brandon Tran, Denada Mertiri, Maarten Lequin, Nilesh K. Desai, Thierry A. G. M. Huisman

**Affiliations:** 1 Edward B. Singleton Department of Radiology, Texas Children’s Hospital, Baylor College of Medicine, 6701 Fannin Street, Suite 470, Houston, TX 77030 USA; 2 Faculty of Medicine and Surgery, Università di Tor Vergata, Rome, Italy

**Keywords:** Cancer predisposition syndromes, Neurofibromatosis type 1, Neurofibromatosis type 2, Von Hippel-Lindau disease, PTEN - hamartoma tumor syndrome, Beckwith-Wiedemann syndrome, Multiple endocrine neoplasia

## Abstract

**Background:**

Cancer predisposition syndromes (CPSs) are inherited disorders that increase the risk of developing cancer from childhood through adulthood. They account for up to 10% of pediatric tumors, making early recognition important for reducing morbidity and mortality. Because these syndromes show variable penetrance and a wide range of clinical presentations even within the same family, identifying affected children can be challenging.

**Main body:**

Imaging is an essential tool for diagnosis, surveillance and follow-up of children with cancer predisposition syndromes. In this review we summarize the main clinical and imaging features of Neurofibromatosis type 1, Neurofibromatosis type 2, von Hippel–Lindau disease, PTEN-hamartoma tumor syndrome, Beckwith–Wiedemann syndrome, and multiple endocrine neoplasia. The goal is to help radiologists and clinicians identify these conditions earlier and improve patient care.

**Conclusion:**

A clear understanding of the clinical and imaging features of cancer predisposition syndromes can support earlier identification, closer surveillance, and improved outcomes. Radiologists play a crucial role in recognizing characteristic patterns and guiding timely management for affected children and their families.

## Introduction

Cancer predisposition syndromes (CPSs) are a group of inherited disorders that significantly increase the lifetime risk of developing cancer. They account for up to 10% of all childhood tumors, making appropriate surveillance essential to reduce morbidity and mortality. Family cancer history, age, and the presence of a specific tumor pattern (tumor type and laterality) should be considered when evaluating a child for a genetic predisposition [1]. In addition to genetic testing, imaging is now an essential tool for the diagnosis, surveillance and follow-up of individuals with suspected or confirmed CPS.

These syndromes show highly variable clinical manifestations, with marked heterogeneity even within families with a shared mutation. CPSs with a particular focus on head and neck imaging findings have been previously described [2]. The purpose of the present article is to complement that prior work by focusing on CPSs that affect multiple organ systems and are frequently encountered in pediatric and adult radiology practice. Specifically, we review the general imaging findings of Neurofibromatosis type 1, Neurofibromatosis type 2, von Hippel-Lindau disease, PTEN- hamartoma tumor syndrome, Beckwith-Wiedemann syndrome, and multiple endocrine neoplasia. We provide a background summary describing genetics and clinical presentations, followed by a detailed description of the imaging findings of the most common associated benign and malignant lesions. CPSs already reviewed in our prior head and neck–focused article [2] are not covered in this article.

## Neurofibromatosis type 1

Neurofibromatosis type 1 (NF1) is an autosomal dominant genetic disorder caused by mutations in the NF1 gene, leading to loss of neurofibromin function and consequent activation of the RAS oncogene [3]. Approximately half of cases are familial, while the remainder result from de novo mutations [4]. Clinical manifestations vary widely even within the same family and include café au lait macules, Lisch nodules (iris hamartomas), learning disabilities, behavior problems, neurofibromas, malignant peripheral nerve sheath tumors (MPNSTs), optic and non-optic gliomas, breast cancer, hematologic malignancies, gastrointestinal stromal tumors (GISTs), rhabdomyosarcomas, pheochromocytomas, paragangliomas and stromal tumors. Affected individuals present with a combination of cutaneous, ocular, neurological, and skeletal findings (Table 1) [3]. In young children, the presence of bony abnormalities, café-au-lait macules, plexiform neurofibromas, and optic gliomas raise the suspicion of NF1 [5].

Neurofibromas are benign peripheral nerve sheath tumors that may arise anywhere along the nerves. Dermal and subcutaneous neurofibromas occur in approximately 60% of NF1 patients [6] and commonly manifest as pedunculated papulonodular cutaneous or subcutaneous lesions in young adults [7]. On ultrasound (US), they appear as well-defined, oval, hypoechoic masses in continuity with a peripheral nerve, and are typically hypovascular on Color Doppler [8].

Plexiform neurofibromas are more complex, firm, superficial or deep masses characterized by longitudinal growth along the nerves, involving multiple fascicles and branches. They may overgrow, infiltrate, and erode adjacent structures [2, 5, 9]. These tumors are pathognomonic for NF1 and occur in 30–50% of patients, usually in infancy or childhood, and most commonly in the craniomaxillofacial region [10, 11]. The ophthalmic branch of the trigeminal nerve is frequently involved and may be associated with sphenoid dysplasia, ductal ectasia and aqueduct stenosis, and buphtalmus [12, 13].

Spinal neurofibromas, in particular those at C2 nerve root, are frequently bilateral and demonstrate intradural extension and myelopathy [10]. Large, multiple paraspinal neurofibromas are often associated with vertebral dysplasia and dystrophic scoliosis [5, 10].

**Fig. 1:**
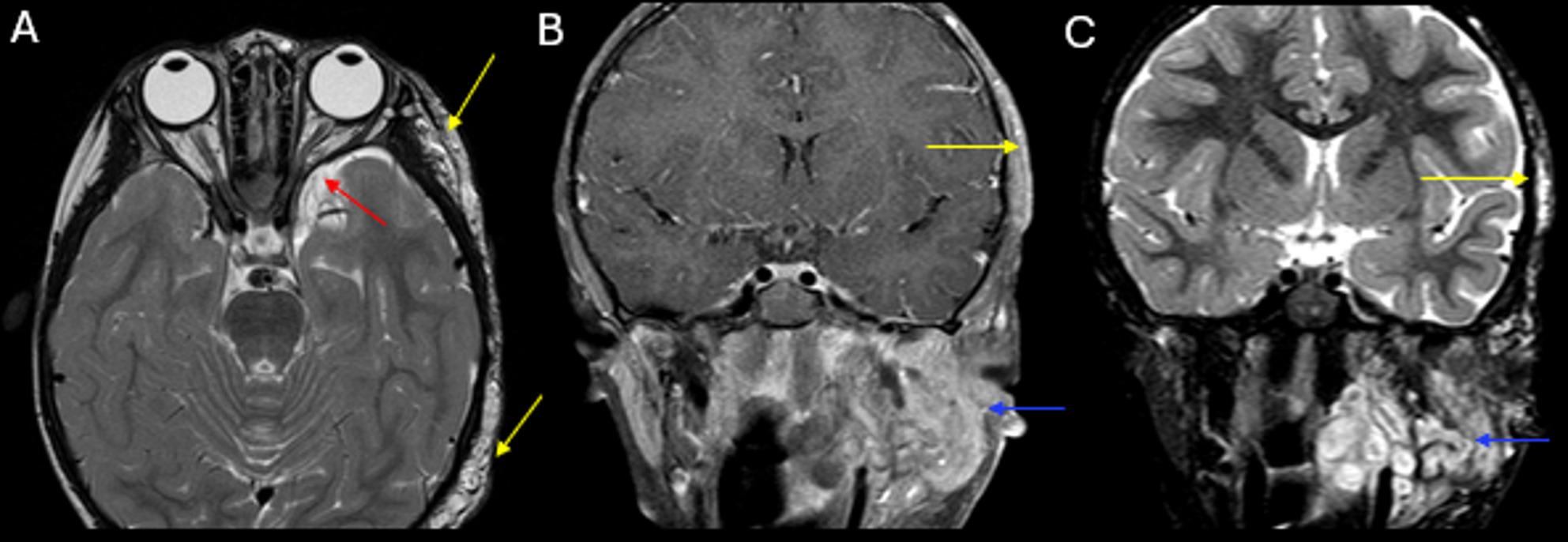
(**A**) Axial T2-weighted image of the orbits demonstrates dysplasia of the left greater wing of the sphenoid (red arrow). Scalp plexiform neurofibromas with hyperintense T2 signal are noted in the left temporal fossa and posterior occipito-temporal convexity (yellow arrows). (**B**) Axial T1 fat-suppressed image and (**C**) coronal STIR image of the sella region show extensive multispatial plexiform neurofibromas centered in the left infratemporal fossa with extension into the parapharyngeal space, masticator space and periauricular region with enhancement and hyperintense STIR (blue arrows in **B** and **C**). There is classic “target sign” within the neurofibroma (blue arrow in **C**)

On MRI, plexiform neurofibromas appear as multinodular, confluent masses with mass effect and the characteristic target sign on T2-weighted images (WI) (hyperintense peripheral rim and hypointense central fibrous component) (Fig. 1). They appear hypointense on T1WI and show variable post-contrast enhancement [5]. Although most plexiform neurofibromas remain benign, the lifetime risk of malignant transformation to MPNSTs is approximately 10% [14]. Malignant progression is thought to occur through stepwise accumulation of genetic alterations, with transition from plexiform neurofibroma to atypical neurofibromatous neoplasm of uncertain biological potential (ANNUBP), and ultimately to MPNST following additional mutations [15]. Currently, ANNUBPs are diagnosed on histopathology; however, imaging plays a key role in identifying lesions suspicious for progression and guiding further evaluation [15, 16]. Imaging features suggestive of malignant transformation include large size, peripheral enhancement, perilesional edema-like signal, intratumoral cystic change, ill-defined margins, and adjacent bone destruction. On ^18^F-FDG PET/CT, a SUVmax > 3.5 favors MPNST, whereas benign lesions typically demonstrate lower uptake [9].Sphenoid wing dysplasia is a hallmark osseous abnormality of NF1 and is characterized by hypoplasia of the greater or lesser sphenoid wing, often demonstrated as an absence of the innominate line on radiographs or CT, known as “bare orbit sign”. This results in widening of the orbital fissures, flattening of the posterior aspect of the orbit, exophthalmos and may be associated with meningocele [9, 17].

Scoliosis is the most common bone abnormality in NF1 patients and may be dystrophic or non-dystrophic. Although less frequent, dystrophic scoliosis typically presents at a younger age (6–10 years vs. adolescence), progresses more rapidly, and is associated with a worse prognosis [9, 18]. It usually involves four to six vertebral bodies, most frequently in the lower cervical/upper thoracic spine, and may be associated with vertebral scalloping, neuroforaminal widening, transverse process spindling [9]. Vertebral body scalloping results from adjacent dural ectasia, neurofibromas, or thoracic meningoceles [9, 18].

Dysplasia of the long bones, especially the tibia and fibula, is a less frequent bone abnormality, but represents a characteristic skeletal manifestation of NF1 in infancy. It is usually unilateral and presents with anterolateral tibial bowing, fractures and pseudoarthrosis due to abnormal bone remodeling [11, 18].

Focal areas of signal intensity (FASI) are a distinguishing imaging finding in children with NF1 and consist of foci of increased T2 and FLAIR signal intensity [10]. They occur in approximately 70% of patients [9], typically appearing by age of 3 years, increasing in number and size until 10–12 years of age [5], and subsequently regressing; they are rarely seen after age of 20years [5, 9, 10]. Pathologically, FASI are considered benign lesions and correspond to spongiform myelopathy and myelin vacuolization [19], though rare cases of tumor development within these areas have been reported, supporting the need for MRI surveillance [20]. FASI are typically located in the cerebellum, brainstem, and basal ganglia, and are non-space-occupying, cause no mass effect or clinical symptoms. However, several studies have demonstrated a strong association between FASI – particularly those in the thalamus and cerebellum - and cognitive impairment [21–23]. On MRI, they appear hyperintense on T2WI and FLAIR, isointense, or occasionally slightly hyperintense on T1WI, and do not enhance after contrast administration. ^1^H-MRS helps differentiate FASI from gliomas, as FASI demonstrate near-normal level of N-acetylaspartate [5, 9, 10].

Optic pathway gliomas (OPGs) are the most common central nervous system (CNS) tumors in NF1, occurring in 15–20% of affected children [24]. Most are asymptomatic, but up to 50% of cases may present with poor visual acuity, optic atrophy, visual field defects or papilledema [5]. OPGs typically manifest in young children with median age of 4 years and may develop at any portion of the optic pathway (e.g., optic nerves, chiasm, optic tracts, lateral geniculate bodies, optic radiations).

**Fig. 2:**
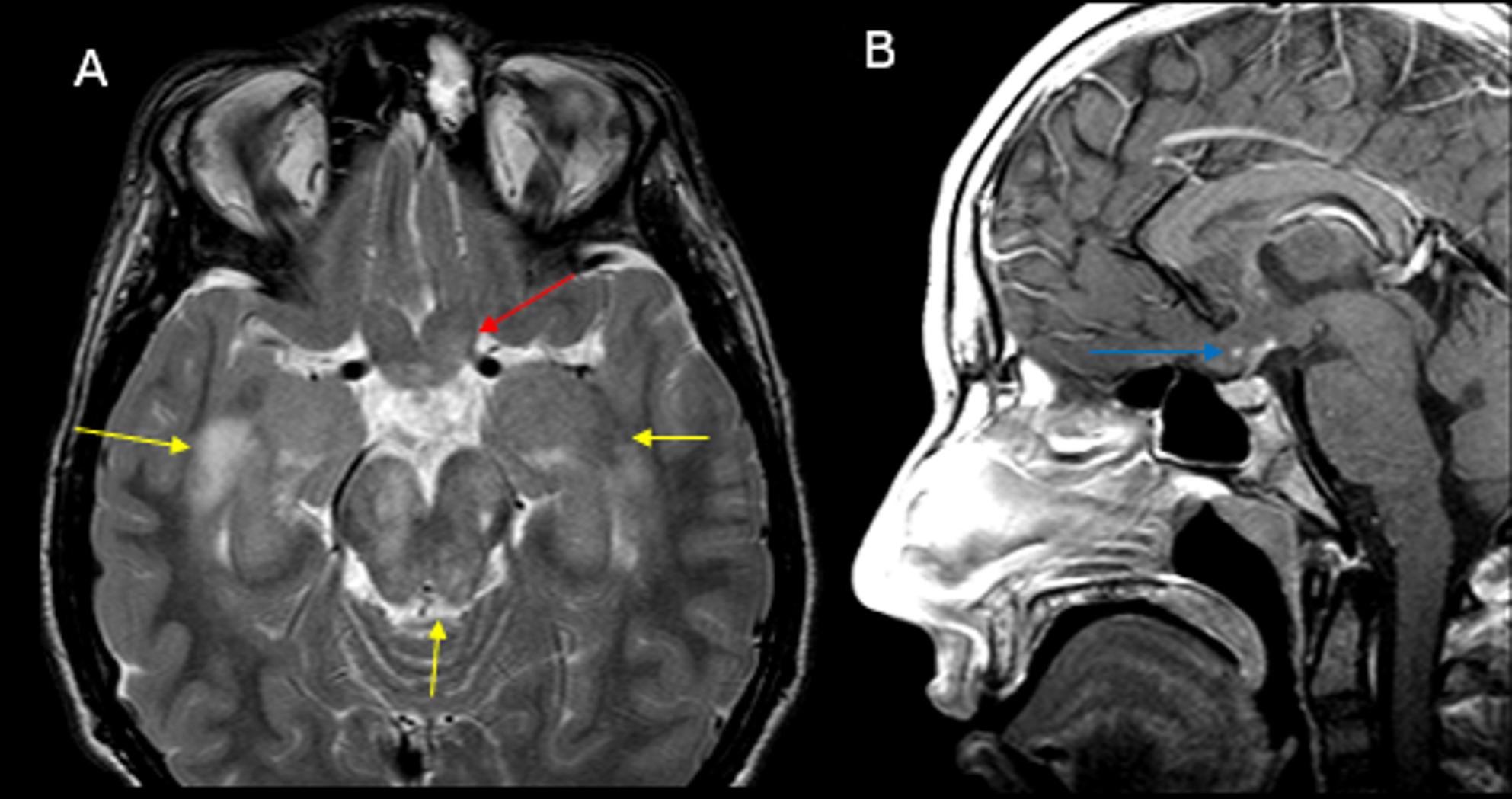
(**A**) Axial T2-weighted image of the orbits shows abnormal thickening of optic chiasm and pre-chiasmatic optic nerves with abnormal hyperintense T2 signal (red arrow), consistent with optic pathway glioma in this NF1 patient. Multifocal areas of spongiform myelin with hyperintense T2 signal are present in the bilateral medial temporal lobes and brainstem (yellow arrows). (**B**) Sagittal T1-weighted image of the sella redemonstrates the optic pathway glioma, showing minimal enhancement in the chiasmatic component (blue arrow in **B**)

MRI of the brain is the modality of choice to detect OPGs and assess their anatomical extent. OPGs cause enlargement of the optic nerve sheath complex that may appear tubular, fusiform, eccentric or globular with tortuosity [5]. They are hypointense on T1WI, hyperintense on T2WI, and usually enhance after contrast administration (Fig. 2) [5, 9, 10]. Fat-saturated sequences improve assessment of optic nerve involvement [5, 25].

Non-optic gliomas occur in approximately 1–2% of NF1 patients and are usually located in the brainstem, cerebellum or spinal cord [5, 10]. Most NF- associated gliomas have a relatively benign course and may even regress [21, 22]. Brainstem gliomas account for approximately 18% of NF1-associated brain neoplasms and occur most commonly in the medulla, followed by the midbrain and pons [5, 26, 27]. Most are asymptomatic, however tectal gliomas may obstruct the aqueduct of Sylvius, leading to hydrocephalus [5, 27]. On MRI, the tectum appears expanded, isointense on T1WI, and hyperintense on T2WI [5]. Cerebellar gliomas are rare in NF1 patients and usually malignant [5].

GISTs occur in up to 25% of individuals with NF1 [8, 9]. In contrast to the general population, in which the stomach is the most common site, NF1-associated GISTs more frequently arise in the small bowel and are often multifocal [8]. On imaging, they appear as submucosal wall masses and may demonstrate exophytic, intraluminal, or mixed growth patterns. Areas of low attenuation within the mass on CT suggest hemorrhage and necrosis [8].

**Table 1:** Diagnostic criteria for neurofibromatosis type 1

- Six or more café-au-lait macules (> 5 mm in children and > 15 mm in adults) - Freckling in the axillary or inguinal regions - Two or more neurofibromas or one plexiform neurofibroma - Optic pathway glioma - Two or more Lisch nodules identified by slit lamp examination or two or more choroidal abnormalities - A distinctive osseous lesion such as sphenoid dysplasia, anterolateral bowing of the tibia, or pseudoarthrosis of a long bone - A parent with NF1 - A germline NF1 pathogenic variant
Diagnosis of NF1 is made when at least two of these features are identified.

## Neurofibromatosis type 2

**Fig. 3:**
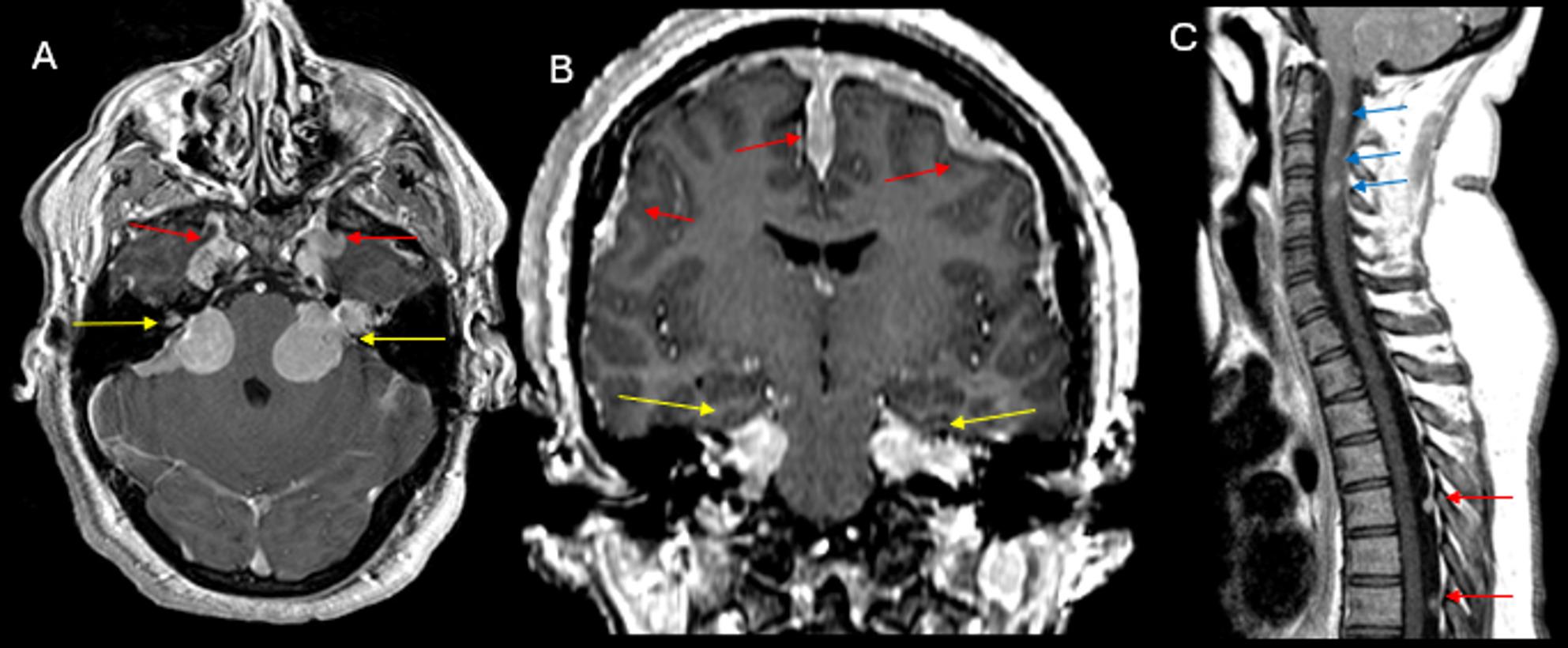
(**A**) Post-contrast axial T1-weighted and (**B**) coronal T1-weighted images demonstrate enhancing bilateral vestibular schwannomas (yellow arrows) and multifocal meningiomas along the cerebral convexity, falx, and bilateral mesial temporal regions (red arrows) in a patient with NF2. (**C**) Post-contrast sagittal T1-weighted image of the cervical and upper thoracic spine shows a few enhancing intramedullary lesions in the cervical cord, suggestive of ependymomas (blue arrows). Additionally, dural based intraspinal extramedullary lesions in the dorsal mid thoracic spine are also present, compatible with meningiomas (red arrows)

Neurofibromatosis type 2 (NF2) is an autosomal dominant syndrome caused by loss-of-function mutations in the NF2 tumor suppressor gene, which encodes merlin, a protein involved in regulating cell growth, particularly in Schwann cells, and cell-cell adhesion [28]. NF2 typically presents in early adulthood and is characterized by multiple inherited schwannomas, meningiomas, and ependymomas (MISME) (Fig. 3). Ophthalmologic manifestations and cutaneous involvement occur in approximately 70% of patients [9], whereas neurofibromas are uncommon [29]. Early age at onset and intracranial meningiomas at presentation are associated with increased mortality [30]. Adults most often present with hearing loss, tinnitus, and balance dysfunction due to bilateral vestibular schwannomas, whereas children more frequently exhibit visual disturbances (e.g., cataracts, optic nerve meningiomas, disk gliomas or retinal hamartomas), skin tumors, mononeuropathies [29, 31, 32].

Schwannomas are benign peripheral nerve sheath tumors and represent the most common neoplasms in NF2. They arise most frequently from the vestibular branch of cranial nerve VIII, and bilateral vestibular schwannomas are pathognomonic for the disorder [9, 28]. Compared with sporadic lesions, NF2-associated vestibular schwannomas are more aggressive, have a higher mitotic index, and a higher risk of recurrence. Disease is typically more aggressive in younger patients [28, 29, 33, 34].

Vestibular schwannomas arise at the neuroglial junction within the internal auditory canal (IAC) and may extend into the cerebellopontine angle if untreated [35]. Large tumors may cause IAC widening, involve the cochlear and facial nerves - resulting in hearing loss or facial paralysis - and compress the brainstem or cerebellum. Severe cases may lead to tonsillar herniation, peritumoral edema, or hydrocephalus [35, 36].

In approximately 50% of patients, NF2-associated schwannomas may affect other cranial nerves, most commonly trigeminal and oculomotor nerves, but may also arise from spinal nerve roots and peripheral nerves [36, 37]. Distinctive plexiform “plaque-like” dermal or subcutaneous schwannomas may occur in NF2, and together with bilateral vestibular schwannomas or ependymomas, help distinguish NF2 from schwannomatosis [38–40].

On CT, schwannomas appear as isoattenuating masses and, unlike meningiomas, vestibular schwannomas usually do not contain calcifications [35]. CT can detect moderate to large lesions, but may miss small intracanalicular tumors [35].

MRI is the preferred diagnostic modality. Brain MRI with thin cuts through the IAC, and in particular sagittal 3D T2WI, provides accurate evaluation of the cochlear nerves [4, 25, 41]. On MRI, schwannomas are hypo- to isointense on T1WI, hyperintense on T2WI, and demonstrate avid post-contrast enhancement [5]. Larger lesions may demonstrate heterogeneous signal due to intralesional hemorrhage, necrosis, or cystic changes [9, 35].

Meningiomas are the second most common tumors in NF2, and the presence of multiple meningiomas (≈ 50% of patients) is a major diagnostic criterion for the syndrome [9, 28]. They most frequently occur supratentorially, particularly in the frontal, parietal, and temporal regions, and along the falx cerebri [9, 28].

On CT, meningiomas appear as hyperdense dural-based lesions with avid contrast enhancement [5, 42]. On MRI, they are isointense to gray matter on both T1 and T2WI, and demonstrate homogeneous enhancement with a dural tail [9]. Adjacent skull hyperostosis or erosion may be present [5, 42]. Spinal meningiomas appear as intradural extramedullary masses [9].

Meningioangiomatosis is a rare, benign, hamartomatous intracranial lesion, with approximately half of cases associated with NF2 [43]. Pathologically, it is characterized by cortical and leptomeningeal meningovascular proliferation with focal calcifications [5, 43]. In NF2, these lesions are often multiple and asymptomatic, whereas sporadic cases typically present with headaches and seizures [5]. They may be single or multiple, intra-axial or extra-axial, and are most commonly located in the frontal and temporal lobes [43]. On CT, meningioangiomatosis appears as a hypodense, round mass with variable calcifications in the cortical or leptomeningeal area, demonstrating minimal or no enhancement [43]. On MRI, lesions are usually iso- to hypointense on T1WI, and heterogeneous with central hypointensity and surrounding hyperintensity on T2WI [5, 44].

Ependymomas are WHO grade 2 lesions and occur in approximately 53% of individuals with NF2 [28]. Patients often develop multiple spinal cord ependymomas, most commonly in the cervical spinal cord or at the cervicomedullary junction, and are usually asymptomatic [28, 45].

On MRI, ependymomas appear as intramedullary tumors, isointense to slightly hyperintense on T1WI and hyperintense on T2WI relative to the normal spinal cord [5, 9]. On post-contrast images, they demonstrate intense enhancement and may assume a characteristic “string of pearls” appearance along the spinal cord or cauda equina [28].

## Von Hippel–Lindau (VHL) disease

Von Hippel-Lindau disease is an autosomal dominant multisystem tumor predisposition syndrome caused by mutations in the VHL tumor suppressor gene, leading to overexpression of proteins that mediate angiogenesis [3, 46]. The phenotype is highly variable in terms of clinical manifestations and age at onset. Patients may develop multiple benign and malignant tumors, including hemangioblastomas of the retina and CNS, renal cell carcinoma (RCC), pheochromocytoma, neuroendocrine tumors, and renal or pancreatic cysts.

**Fig. 4:**
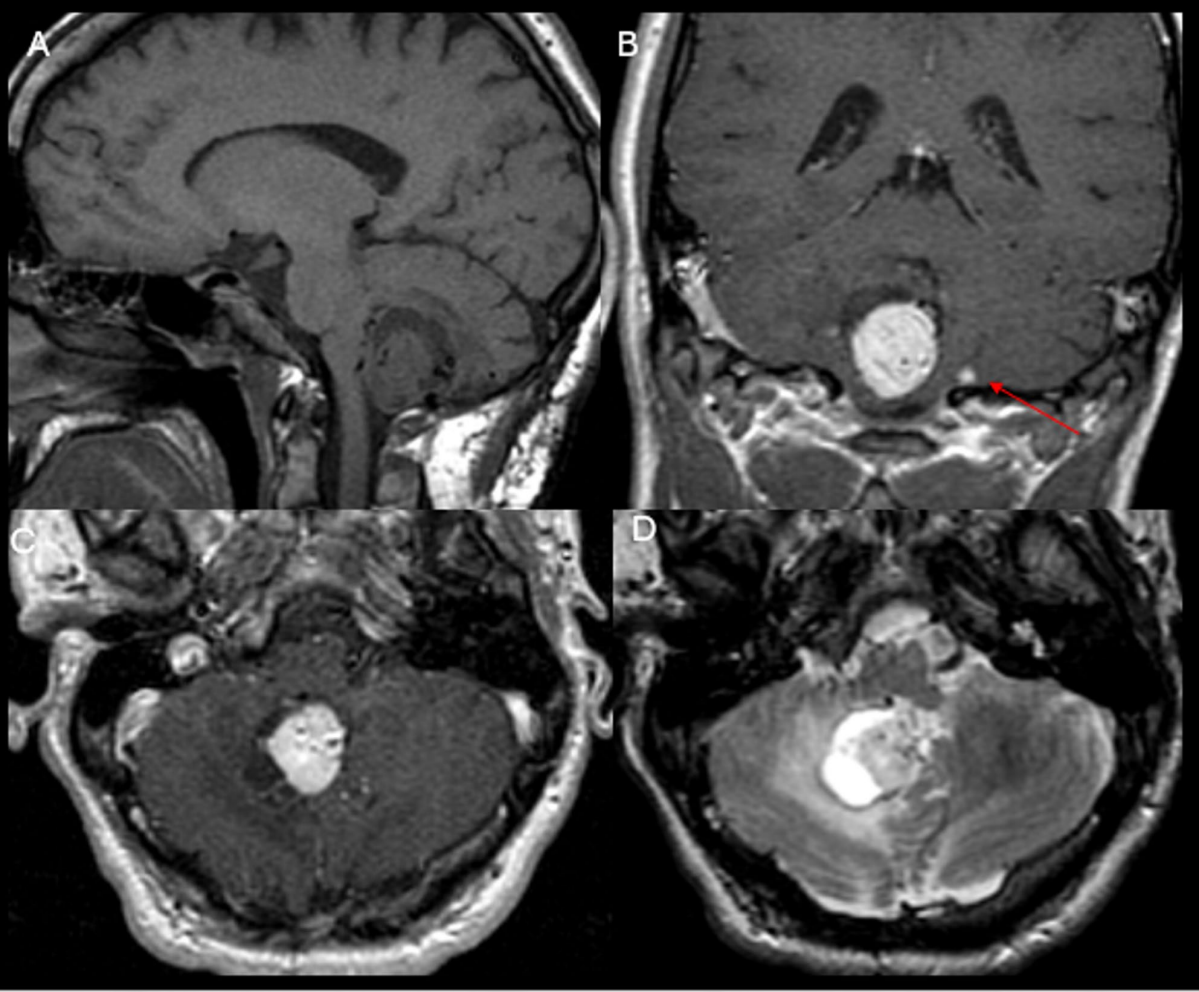
(**A**) Pre-contrast sagittal T1-weighted image (WI), (**B** and **C**) post-contrast coronal and axial T1WI and (**D**) Axial T2WI. There is a mild enhancing intra-axial mass in the right paramedian inferior cerebellum with associated small cysts and associated perilesional vasogenic edema demonstrating T2 hyperintensity. In addition, a small homogeneously enhancing nodule is present in the inferior aspect of the left cerebellum (red arrow in B). Multiple dilated vessels are noted along the tumor secondary to the characteristic high vascularity of the tumor. These findings are consistent with hemangioblastomas

CNS hemangioblastomas are benign WHO grade 1 tumors characterized by a rich capillary network [47]. They form cysts of various sizes, are often multifocal, and occur most commonly in the cerebellum (≈ 60%), followed by the spinal cord and brainstem [47, 48]. The mean age of onset is 33 years [48]. Symptoms are usually caused by the associated cysts or syrinx rather than the tumor itself, as these cystic components tend to grow faster and become larger [49]. On CT, hemangioblastomas appear as well-defined, homogeneous hypodense cysts with an avidly enhancing mural nodule within the cyst wall [48, 50, 51]. On MRI, the cystic component is typically T1-hypointense and T2-hyperintense, although signal intensity may vary depending on protein content or the presence of hemorrhage (Fig. 4) [50, 52]. The solid tumor component shows high T2 signal, abuts the cerebellar surface, and demonstrates marked enhancement on post-contrast T1WI, while the cyst wall does not enhance [48, 50, 51].

Because spinal lesions frequently coexist, spinal MRI should be obtained whenever a cerebellar hemangioblastoma is identified [48, 53]. Close surveillance is recommended, as many tumors exhibit a saltatory growth pattern [53].

The differential diagnosis includes metastases, medulloblastomas, pilocytic astrocytomas, and ependymomas due to overlapping imaging features. Metastases are usually located at the gray-white matter junction of the supratentorial brain, show ring enhancement, and are usually multiple [51]. Pilocytic astrocytomas are typically less vascular and lack flow voids; dynamic susceptibility contrast perfusion-weighted imaging and MR spectroscopy may help in differentiation [54]. Medulloblastomas more frequently arise from the midline cerebellar vermis and present with marked peritumoral edema and restricted diffusion [55]. Ependymomas usually extend from the fourth ventricle into adjacent cisterns and often contain calcifications [55].

**Fig. 5:**
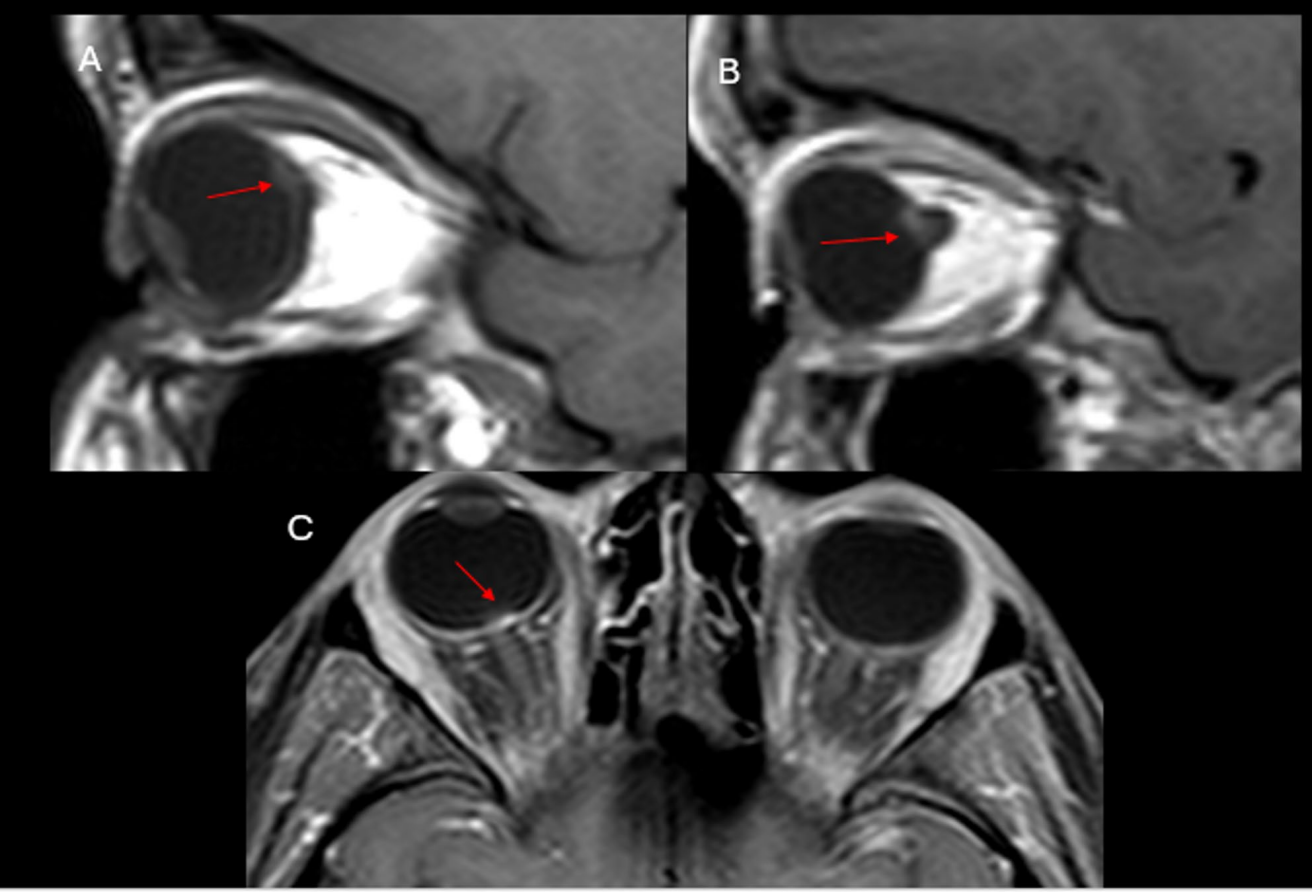
(**A**) Pre-contrast sagittal T1-weighted image (WI), (**B**) post-contrast sagittal T1WI, and (**C**) post-contrast axial T1-fat saturated (**C**) of the orbits demonstrate a small enhancing lesion in the nasal aspect of the right optic disc. It is consistent with retinal hemangioblastoma in this patient with history of Von Hippel Lindau syndrome

Retinal hemangioblastomas develop in 45–60% of VHL patients and the mean age of onset is 25 years [48, 56, 57]. Sometimes they may be the only manifestation of the disease, and in half of the cases are bilateral [48, 57]. On fundoscopy, they have a globular appearance with dilated, tortuous feeding vessels and optic disc edema, and in 85% of cases they occur in the peripheral retina [58]. The diagnosis is confirmed on ophthalmic examination. Imaging plays a limited role in the diagnosis of retinal hemangioblastomas; however, brain MRI may show an enhancing lesion with or without retinal detachment (Fig. 5) [48, 59].

Endolymphatic sac tumors are benign but locally invasive neoplasms of the vestibular aqueduct, occurring in approximately 10–15% of individuals with VHL [48]. The mean age at presentation is 22 years, and tumors may be bilateral [48]. Clinical manifestations include hearing loss, tinnitus, vertigo, and facial nerve palsy [48]. On CT, they demonstrate a moth-eaten appearance of the petrous temporal bone with erosions involving the vestibular aqueduct, semicircular canals, and cochlea [60, 61]. Central calcific spicules and posterior rim calcifications are frequently noted [60]. On MRI, they appear hyperintense on both T1- and T2WI due to hemorrhagic and proteinaceous contents, with marked enhancement after gadolinium [48, 60, 61]. Angiography may be useful to assess intracranial vessel involvement and carotid artery infiltration before surgery [62].

Renal cysts and clear cell RCC occur in more than two-thirds of patients with VHL [48]. Renal cysts are usually bilateral and multiple, and their malignant potential depends on size and number [48, 63]. Cysts may be simple or complex (containing both cystic and solid components). US can help differentiate solid from cystic lesions; however, CT and MRI are preferred for further assessment. On CT, simple cysts appear as thin-walled lesions with fluid density with no or minimal enhancement. On MRI, they show homogeneous T1 hypointensity, T2 hyperintensity, and lack post-contrast enhancement [48, 63]. Complex cysts are precursors to RCC [63]. Solid RCCs are usually heterogeneous and demonstrate avid early enhancement followed by washout in the delayed phase [48]. On MRI, they are T1-hypointense, T2-hyperintense, and show marked enhancement. Cystic RCCs exhibit enhancing nodular components and/or thick nodular septa [48].

RCC is the most common cause of mortality in patients with VHL, and the presence of bilateral or multifocal RCC in individuals younger than 50 years should prompt VHL genetic testing [64]. VHL-associated RCCs occur at a younger age than sporadic RCCs, with a mean age of 39 years [48].

Adrenal pheochromocytomas develop in 25–30% of patients with VHL [48]. The mean age of onset is 27 years, and in 50% of cases they are bilateral [65]. Extra-adrenal locations along the sympathetic chain in the abdomen, thorax, or head and neck have been seen in 15% of VHL cases [48, 63]. On CT, pheochromocytomas appear as solid or complex cystic masses with areas of necrosis and hemorrhage, calcifications, and marked enhancement [63]. However, CT enhancement characteristics may not distinguish them from other lesions that may show similar enhancement (adenomas, or hypervascular adrenal metastases) [66]. On MRI, they typically show iso- or hypointensity relative to the liver on T1WI and on T2WI they appear as a ‘light-bulb’ bright lesion comparable to the signal intensity of cerebrospinal fluid [66, 67]. ^123^I-metaiodobenzylguanidine (MIBG) scintigraphy may be useful in tumor localization, although PET/CT offers higher sensitivity for detecting metastatic disease [68, 69]. The diagnosis of pheochromocytoma is confirmed on biochemical tests (serum and urinary catecholamines) [63].

Pancreatic manifestations in patients with VHL include cysts, serous cystoadenomas, and pancreatic neuroendocrine tumors (NETs). Pancreatic cysts are usually multiple, asymptomatic, and may represent the only manifestation at the time of initial diagnosis in up to 12% of patients [70]. On CT, they appear as hypoattenuating lesions with fluid attenuation and without enhancement [48]. Serous cystoadenomas present as multilobulated cystic masses with a characteristic grape appearance on CT. An enhancing central scar with stellate calcification may be seen in 20% of cases [48]. On MRI, the central scar may appear hypointense on both T1 and T2WI with delayed enhancement. Pancreatic NETs are usually hypo- to isointense on CT and exhibit marked early arterial enhancement, sometimes with necrotic areas and heterogeneous enhancement in larger lesions [63]. Tumors smaller than 3 cm are usually solid and homogeneous. On MRI, they appear T1-hypointense and T2-hyperintense with avid arterial enhancement, with washout on delayed phase images [48].

## PTEN hamartoma tumor syndrome – Cowden syndrome

Cowden Syndrome (CS) is a rare cancer predisposition syndrome caused by autosomal dominant mutations in the PTEN tumor suppressor gene [71]. It is part of the PTEN hamartoma tumor syndrome (PHTS) family, which predisposes affected individuals to the development of hamartomas and an increased risk of benign and malignant tumors, particularly of the thyroid, breast, kidney, colon, and endometrium [71, 72]. Mucocutaneous lesions, observed in nearly all patients, are usually the earliest clinical manifestation and include papules, papillomas, trichilemmomas, and acral keratoses [73]. Other non-typical cutaneous lesions include angiomas, lipomas, neurinomas, xanthomas, melanomas, squamous cell carcinoma, and basal cell carcinoma [71, 73]. In addition to cancer susceptibility and dermatologic findings, PHTS features include Lhermitte-Duclos disease, macrocephaly, vascular anomalies, and autism spectrum disorder [74–76]. Intracranial meningiomas have also been reported in the setting of CS, although this association remains unconfirmed [77, 78]. The National Comprehensive Cancer Network diagnostic criteria (Table 2) are currently used for diagnosing CS in individuals older than 18 years [74]. Bannayan-Riley-Ruvalcaba syndrome (BRRS) is considered a phenotypic form of CS in children and is characterized by macrocephaly, intestinal hamartomatous polyposis, lipomas, and pigmented macules of the glans penis [74, 75].

Lhermitte-Duclos Disease (LDD) is a benign dysplastic gangliocytoma of the cerebellum, usually diagnosed at approximately 20–30 years of age [74]. It usually affects a single cerebellar hemisphere, with a slight right-sided predilection; the vermis may also be involved [79]. Patients typically present with ataxia, nystagmus, and increased intracranial pressure [80]. On MRI, LDD appears as a T2/FLAIR hyperintense cerebellar lesion with widening of the cerebellar folia, producing the characteristic “tigroid” appearance [81]. In children, however, diagnosis may be challenging because the typical “tiger stripe” pattern may be absent [82] and childhood-onset LDD is less frequently associated with PTEN mutations [74]. Although LDD is usually described as non-enhancing, linear or dot-like enhancement, as well as curvilinear enhancement secondary to small draining veins, has been reported [83–86]. MRI is also valuable for evaluating posterior fossa mass effect caused by LDD, as well as secondary obstructive hydrocephalus and cerebellar tonsillar herniation [87, 88]. Downward herniation of the cerebellar tonsils has been described in several cases and it is important to determine whether it is acquired or developmental, as it may also occur in the absence of LDD [83].

**Fig. 6:**
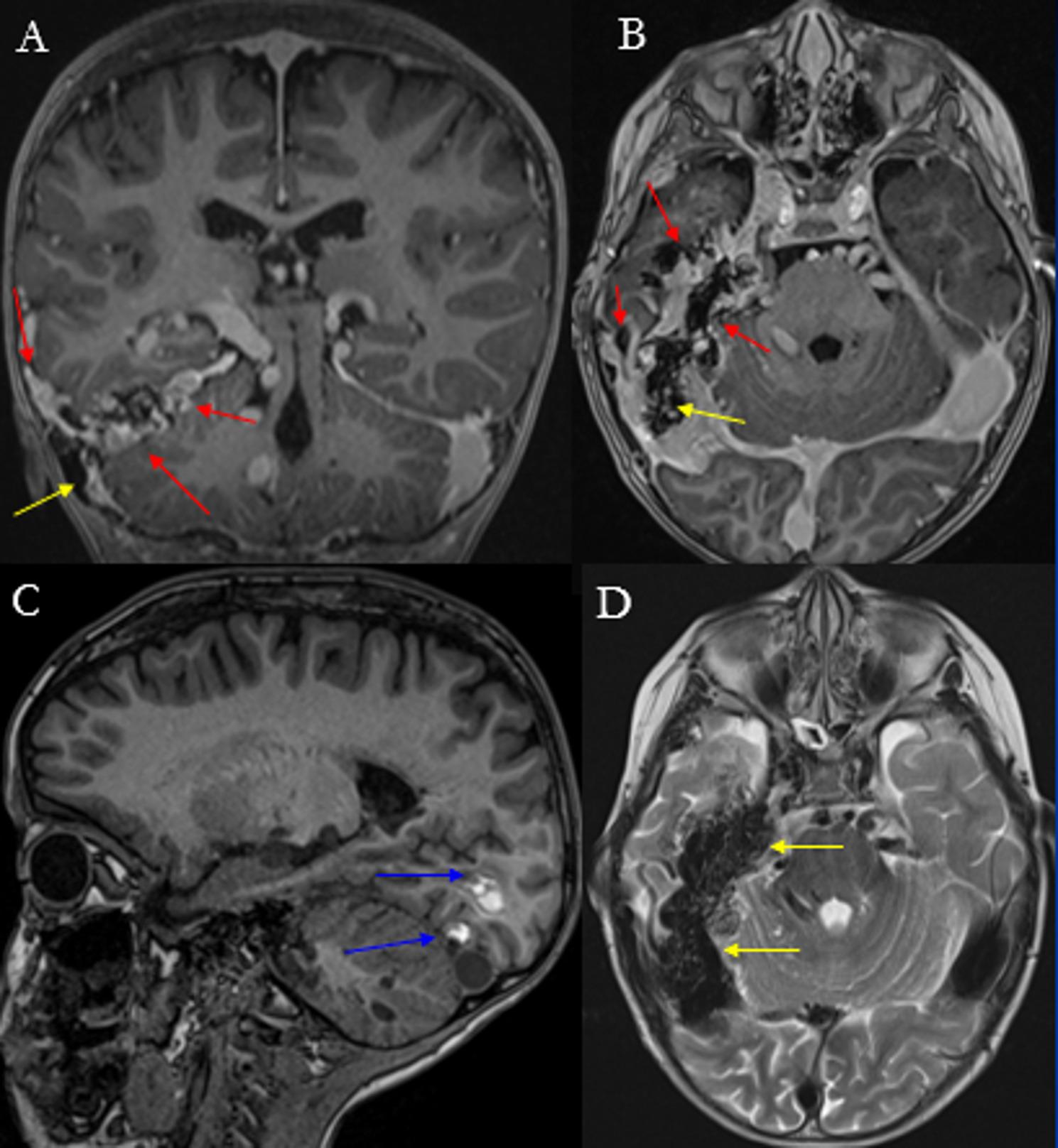
Child with PTEN hamartoma tumor syndrome/Cowden syndrome. (**A** and **B**) Post-contrast coronal and axial T1-weighted image (WI), (**C**) pre-contrast sagittal T1WI, and (**D**) axial T2WI. There are many small dural fistulas (in the floor of the right middle cranial fossa, posterior fossa and along the tentorial leaflet with curvilinear vascular enhancement due combination of feeding arterial branches from posterior cerebral artery and inferior middle cerebral artery division branches and draining veins (red arrows in **A** and **B**) in conjunction with dilated dural sinuses. Post embolization changes with hypointense coiling materials are also noted (yellow arrows in **A**, **B** and **C**). Additionally, two small cavernomas with hyperintense T1 subacute hemorrhage are present in the occipital lobe on the right (blue arrows in **C**)

Vascular malformations, including arteriovenous malformations and hemangiomas, are frequently reported in BRRS and CS patients and represent a minor diagnostic criterion for PHTS [74]. They present as multifocal, intramuscular, or intracranial developmental abnormalities [81] (Fig. 6).

Macrocephaly in PHTS is typically due to generalized megalencephaly, characterized by brain overgrowth resulting from an increased number or size of neurons and glial cells. In most patients, it is symmetrical, although cases with hemimegalencephaly have also been described [80, 89]. Brain MRI should also be assessed for other cortical malformations, including focal cortical dysplasia, polymicrogyria, and periventricular nodular heterotopia, all of which have been reported in children with PTEN mutations [90–92].

Breast cancer is the most common tumor in patients with CS. On MRI, breast lesions typically demonstrate enhancement on post-contrast images, often with irregular margins and suspicious washout or plateau enhancement kinetics; however, they do not demonstrate specific imaging features that differentiate them from sporadic cases [81].

Thyroid carcinoma is the second most common cancer in patients with CS with an estimated risk of 3–10% [81]. Thyroid benign lesions are also very common beginning in childhood and include nodular goiter, single or multiple adenomas, autoimmune thyroiditis [80]. US is the preferred initial imaging modality and helps differentiating benign from malignant lesions. Solid hypoechoic nodules with irregular margins, absence of a hypoechoic halo surrounding the nodule, internal vascularity, and possible microcalcifications are suggestive of malignancy. There are no imaging differences between CS-associated thyroid cancer and those in the general population [81].

**Table 2:** Clinical diagnostic criteria for Cowden syndrome

Major criteria	Minor criteria
1. Breast cancer	1. Autism spectrum disorder
2. Endometrial cancer	2. Colorectal cancer
3. Follicular carcinoma of the thyroid gland	3. Esophageal glycogenic acanthosis (≥ 3)
4. Gastrointestinal hamartomas (including ganglioneuromas, but excluding hyperplastic polyps; ≥3)	4. Lipoma (≥ 3)
5. Adult-onset Lhermitte-Duclos disease	5. Intellectual disability (IQ ≤ 75)
6. Macrocephaly (> 97th percentile: 58 cm in women and 60 cm in men)	6. Renal cell carcinoma
7. Macular pigmentation of the glans penis	7. Testicular lipomatosis
8. Multiple mucocutaneous lesions (any of the following): - Acral keratoses (≥ 3, palmoplantar keratotic pits and/or acral hyperkeratotic papules) - Multiple trichilemmomas (≥ 3, at least one biopsy proven): - Mucocutaneous neuroma (≥ 3)	8. Thyroid cancer (papillary carcinoma or follicular variant of papillary)
	9. Thyroid structural lesions (adenoma, adenomatous goiter, etc.)
	10. Vascular anomalies (e.g., multiple developmental venous anomalies)

## Beckwith-Wiedemann syndrome (BWS)

**Fig. 7:**
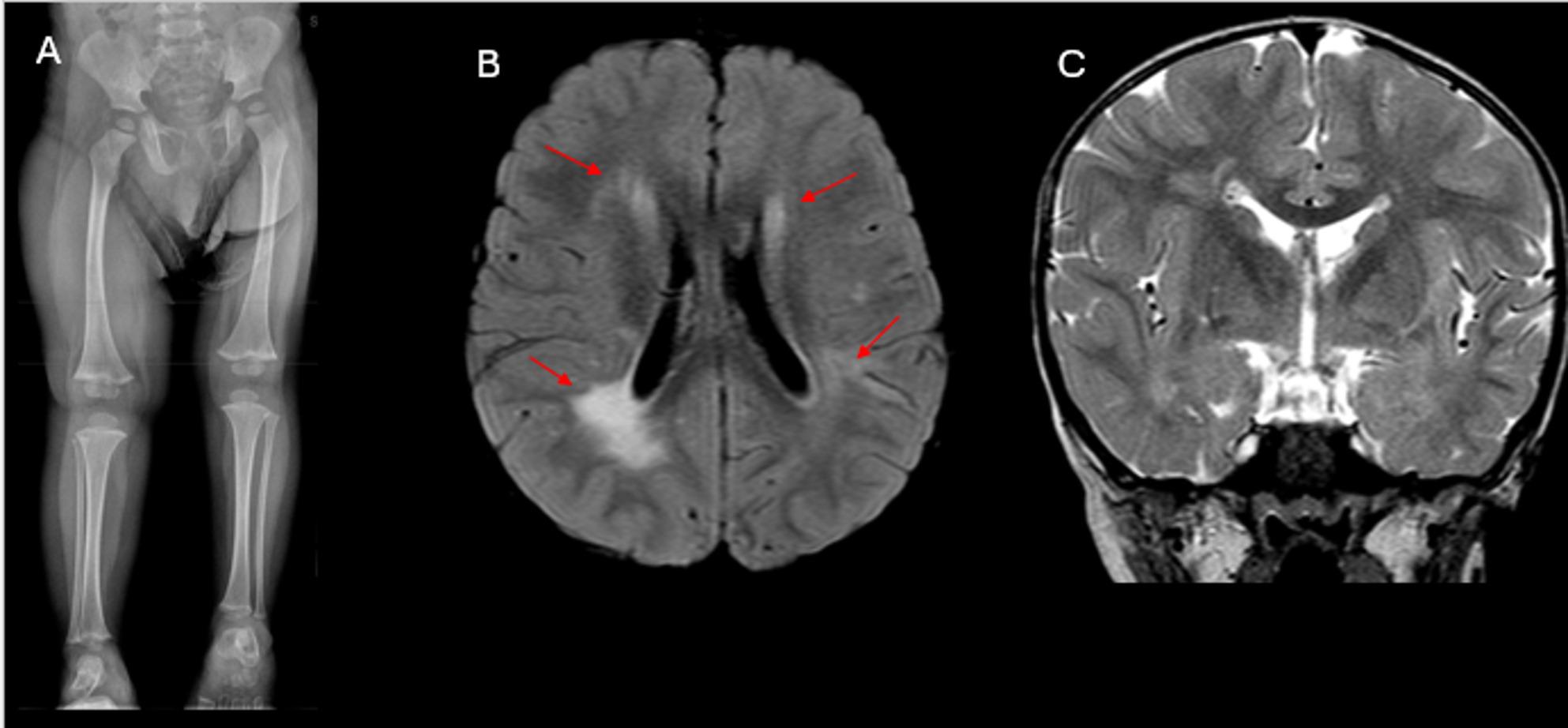
Child with Beckwith-Wiedemann syndrome. (**A**) Leg length radiograph demonstrates leg length discrepancy, with left lower extremity shorter than the right, and hypertrophy of the right lower extremity. (**B**) Axial FLAIR and (**C**) coronal T2-weighted images show asymmetric enlargement of the right cerebral hemisphere, consistent with right hemimegalencephaly. Additionally, abnormal T2 and FLAIR hyperintense gliosis is present in the periventricular white matter bilaterally (red arrows **B**)

Beckwith-Wiedemann syndrome is a rare multisystem overgrowth disorder caused by genetic and epigenetic defects affecting the chromosome 11p15.5 region [93, 94]. This locus contains several imprinted genes, including CDKN1C and IGF2, which play key roles in fetal growth regulation [93, 94]. BWS is typically diagnosed in the neonatal period or early childhood and is clinically characterized by macroglossia, macrosomia, abdominal wall defects, hemihyperplasia, hemimegalencephaly, and severe neonatal hypoglycemia (Fig. 7) [93]. Additional features include exophthalmos, hypertelorism, nystagmus, infraorbital creases, facial nevus flammeus, midfacial hypoplasia, full lower face with a prominent mandible, anterior earlobe creases, and posterior helical pits [94] (Table 3).

Tumor risk is higher during the first decade of life, with a marked predisposition to embryonal tumors, particularly Wilms tumor (WT) and hepatoblastoma [95]. Less frequently, rhabdomyosarcoma, adrenocortical carcinoma, and neuroblastoma have also been reported [95]. An increased tumor risk has been observed in patients with hemihyperplasia or nephromegaly [96].

WT is the most frequently reported tumor in patients with BWS, occurring in approximately 1–8% of affected individuals [97]. Compared with sporadic cases, BWS-associated WTs tend to present earlier (typically before 2 years of age), are often bilateral or multifocal, and frequently coexist with nephrogenic rests (NRs), persistent embryonal tissue representing WT precursors [98, 99]. Abdominal US is usually the initial imaging modality for suspected WT, and prenatal US, particularly in the presence of an omphalocele, may enable early diagnosis of BWS [100, 101]. On US, WTs typically appear as intrarenal masses with a pseudocapsule, often containing large hypoechoic areas reflecting central necrosis, hemorrhage, and cyst formation [102]. On MRI, the ‘claw sign’, in which the normal renal parenchyma stretches and wraps around the mass, is indicative of WT [103, 104]. These tumors are generally hypointense on T1WI and demonstrate variable-to-high signal intensity on T2WI, with noncystic components typically showing diffusion restriction. In contrast to neuroblastoma, vessels are displaced rather than encased, and vascular invasion occurs in approximately 5–10% of cases [102, 103]. NRs are observed in up to 70–80% of patients with BWS-associated WT and are usually located at the periphery of the renal cortex (perilobar NRs) [98, 105]. Post-contrast T1WI can help differentiate NRs or nephroblastomatosis (multiple or diffusely distributed NRs) from WTs, as these lesions show little or no enhancement compared with WTs [104].

Hepatoblastomas are large, well circumscribed neoplasms and occur in approximately 8–14% of patients with BWS [106]. On US, they are hyperechoic relative to the adjacent liver, though echogenicity may vary depending on the histologic type [106, 107]. Mixed tumors appear heterogeneous with calcifications causing echogenic shadowing, and anechoic foci reflecting hemorrhage and necrosis; hypoechoic septa may also be present. On CT, they are hypoattenuating compared to normal liver parenchyma and demonstrate mild post-contrast enhancement. On MRI, lesions are usually hypointense on T1WI and hyperintense on T2WI, with diffusion restriction in the solid components of mixed tumors [106, 107].

**Table 3:** Clinical features of Beckwith–Wiedemann syndrome

**Major Findings:**
• Abdominal wall defect: omphalocele or umbilical hernia • Macroglossia • Macrosomia* (defined as height and weight > 97th percentile) • Anterior ear lobe creases and/or posterior helical pits (bilateral or unilateral) • Visceromegaly of intra-abdominal organ • Embryonal tumor in childhood • Hemihyperplasia • Cytomegaly of adrenal fetal cortex, usually diffuse and bilateral • Renal abnormalities, including medullary dysplasia and later development of medullary sponge kidney • Positive family history of BWS • Cleft palate
**Minor findings**
• Polyhydramnios, enlarged placenta and/or thickened umbilical cord, premature birth • Neonatal hypoglycemia • Nevus flammeus • Cardiomegaly/structural cardiac anomalies/cardiomyopathy • Characteristic facies • Diastasis recti • Advanced bone age

## Multiple endocrine neoplasia

Multiple endocrine neoplasia (MEN) is a group of autosomal dominant disorders that predispose affected individuals to the development of benign and malignant endocrine and neuroendocrine tumors (Table 4) [108]. MEN1 is caused by mutations in the MEN1 tumor suppressor gene and is characterized by tumors of the parathyroid glands (95%), pancreas (40%), and pituitary gland (30%) [108, 109]. Additional manifestations include angiofibromas, collagenomas, adrenal cortical tumors, and facial ependymomas [109]. MEN 2 includes MEN2A, MEN2B, and familial medullary thyroid carcinoma (FMTC) and results from mutations in the RET proto-oncogene [3]. The hallmark tumors of MEN2 include medullary thyroid carcinoma (MTC), adrenal pheochromocytomas, and parathyroid tumors.

Parathyroid adenomas are the most common tumors and usually the presenting feature of MEN1 [108, 110]. Hyperparathyroidism occurs in more than 90% of patients, typically between 20 and 25 years of age [109]. In contrast to sporadic cases, where a solitary adenoma is present in 90% of patients, individuals with MEN1 demonstrate asymmetric multiple glands disease [110]. MEN2A is also associated with multiglandular involvement, but it usually has a later onset and occurs in the setting of concomitant MTC [110, 111].

On US, parathyroid adenomas appear as well-defined, oval hypoechoic masses posterior to the thyroid gland. Large adenomas may appear multilobulated and contain echogenic foci [110, 112]. Color and power Doppler demonstrate a prominent feeding artery that courses along the periphery before entering the gland, producing a characteristic arc or rim of vascularity [112]. Focal asymmetric hypervascularity of the overlying thyroid gland may further assist localization of the underlying adenoma [112].

CT provides no additional information but is helpful for identifying abnormal ectopic glands within the mediastinum or behind the trachea.

On single-photon emission computed tomography sestamibi imaging, adenomas show asymmetric focal radiotracer uptake with retention on delayed imaging, distinguishing them from normal tissue [108, 112].

Parathyroid adenomas are typically hypointense on T1WI and hyperintense on T2WI, though MRI is not usually used for diagnosis [110].

Anterior pituitary adenomas occur in approximately 30% of MEN1 patients [110]. Up to 60% of the hormone-secreting tumors are prolactinomas, followed by growth hormone-secreting tumors (< 25%), and, rarely, adrenocortical hormone- or thyroid stimulating hormone- secreting tumors [113]. Non-functioning adenomas account for about 5% [108].

**Fig. 8:**
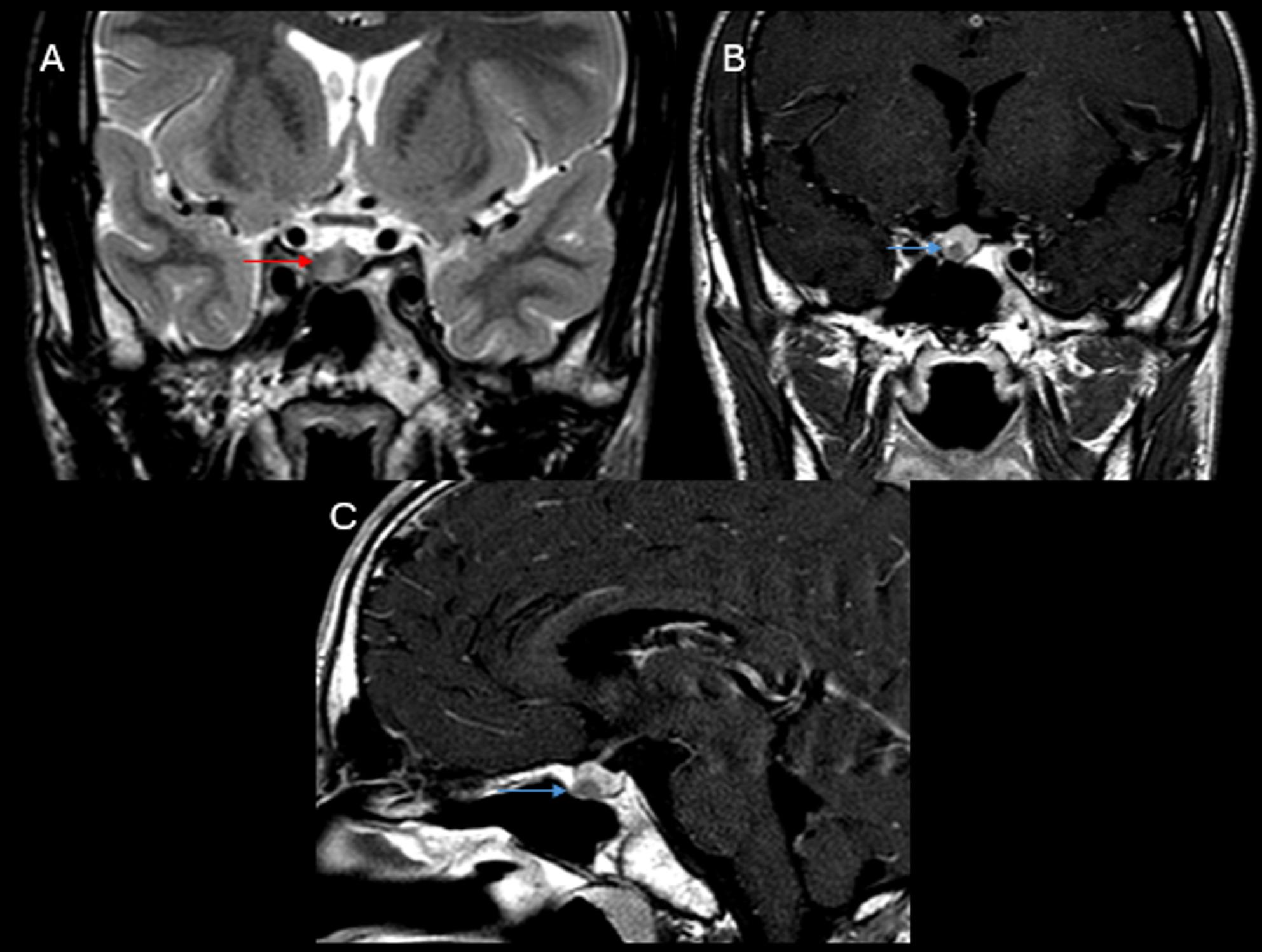
Patient with multiple endocrine neoplasia 1 with recurrent microadenoma. (**A**) Coronal T2-weighted image (WI), and post-contrast coronal (**B**) and sagittal (**C**) T1WI demonstrate a small well-circumscribed hypoenhancing nodule in the inferior adenohypophysis near the floor of the sella turcica (blue arrows in **B** and **C**). The lesion is hyperintense on T2 WI (red arrow in **A**), consistent with small pituitary microadenoma. There is no involvement of the cavernous sinuses or carotid arteries

Small-field-of-view MRI with ≤ 3 mm slice thickness pre- and post-contrast T1WI of the sella is the imaging modality of choice [110]. Adenomas typically appear hypointense relative to the normal pituitary tissue on both pre- and post-contrast T1WI (Fig. 8). Microadenomas (< 10 mm) may not always be visualized on imaging; however, secondary signs – such as focal convexity of the superior pituitary gland, deviation of the pituitary stalk or lowering/erosion of the sellar floor - may suggest the diagnosis. Dynamic contrast-enhanced MRI may improve contrast between microadenomas and normal pituitary tissue by evaluating enhancement on early- and delayed-phase images [114]. Microadenomas typically show less contrast enhancement than the normal pituitary gland tissue on the early arterial phases with persistent contrast enhancement on the late phase images. Macroadenomas (> 10 mm) are usually isointense to gray matter on T1WI, however, attenuation and signal characteristics vary significantly depending on tumor components such as hemorrhage, cystic transformation, or necrosis [113]. Mass effect on adjacent structures is common [108].

Pancreatic NETs occur in approximately 80% of MEN1 patients and may be functioning or nonfunctioning [108]. Gastrinomas are the most common (60%), followed by insulinomas (30%); glucagonomas, VIPomas, and somatostatinomas represent less than 5% [111, 115].

Transabdominal US has low sensitivity for detecting pancreatic NETs. Endoscopic US is much more sensitive but invasive and limited to specialized centers [110]. Intraoperative US is highly effective, typically showing solid hypoechoic masses with occasional hypo- to anechoic cystic foci and calcified components [113].

CT is the most widely used modality for localization [110]. NETs appear as small (<2 cm), often multiple, arterial-phase–enhancing lesions, isodense to the pancreas on non-contrast images. CT is also useful for evaluating local invasion or liver metastases [108, 110].

MRI offers superior sensitivity for small lesions. NETs are typically T1-hypointense and T2-hyperintense, although T2 signal may be reduced in highly collagenous tumors [108, 110]. Liver metastases often display early arterial peripheral ring enhancement [110, 113].

Somatostatin receptor imaging with ^111^ In-octreotide helps detect NETs with somatostatin receptors. ^18^ F-FDG PET may identify aggressive NETs that typically lack somatostatin receptor expression, but demonstrate hypermetabolic activity [110].

Adrenal cortical adenomas occur in up to 40% of MEN1 patients and are usually nonfunctioning [116]. CT or MRI are diagnostic. Up to 80% are lipid-rich, showing very low CT attenuation (< 10 HU), and signal dropout on opposed-phase T1W gradient recalled echo images [117]. Adrenal cortical carcinomas are very rare, but present as large heterogeneous masses with central necrosis, hemorrhage, and calcification, with potential local invasion and distant metastases [108].

MTC is multicentric, bilateral, and occurs in nearly all MEN2 patients. It is aggressive and may metastasize to the liver, lungs, bones, and brain. Serum calcitonin and carcinoembryonic antigen levels are usually elevated. On US, CT, and MRI, MTC appears as a solid mass often containing calcifications. Whole-body radionuclide imaging with ^123^I-MIBG, pentavalent ^99m^Tc-dimer-captosuccinic acid, and somatostatin analogues such as ^111^In-octreotide allows accurate staging [110].

Pheochromocytomas occur in up to 50% of MEN2 patients and are bilateral in most cases [118]. Biochemical screening with urinary metanephrines and imaging is essential before thyroid surgery to reduce perioperative morbidity and mortality [111].

On CT, lesions typically have attenuation > 10 HU and show avid enhancement after contrast administration [119]. CT has near-100% sensitivity, but MRI is preferred due to lack of radiation and the characteristic “light-bulb bright” T2 hyperintensity [108].

^18^F-DOPA PET/CT is recommended as first-line imaging for patients with inherited forms of pheochromocytoma, whereas ^123^I-MIBG may be considered first line in sporadic cases [120].

**Table 4:** Clinical features of MEN syndromes

MEN1	MEN2A	MEN2B	FMTC
Parathyroid tumors	Medullary thyroid carcinoma	Medullary thyroid carcinoma	Familial medullary thyroid carcinoma
Pancreatic islet cell tumors	Pheochromocytoma	Pheochromocytoma	
Pituitary tumors	Parathyroid tumor	Associated abnormalities: - Mucosal neuromas - Marfanoid habitus - Multiple ganglioneuromas	
Other tumors: - Angiofibromas - Collagenomas - Adrenal cortical tumors - Carcinoid tumors			

## Conclusion

Imaging plays a central role in the evaluation of patients with CPSs. Given the wide spectrum of benign and malignant tumors associated with these disorders, imaging assessment—together with genetic testing—is essential for both diagnosis and surveillance. Familiarity with characteristic imaging patterns enables early tumor detection and guides timely clinical management, ultimately improving outcomes for individuals with suspected or confirmed CPS.

## Data Availability

No datasets were generated or analysed during the current study.

## Abbreviations

BRRS: Bannayan-Riley-Ruvalcaba syndrome
BWS: Beckwith-Wiedemann syndrome
CNS: Central nervous system
CPS: Cancer predisposition syndrome
CS: Cowden syndrome
FASI: Focal areas of signal intensity
FMTC: Familial medullary thyroid carcinoma
IAC: Internal auditory canal
LDD: Lhermitte-Duclos disease
MEN: Multiple endocrine neoplasia
MIBG: Metaiodobenzylguanidine
MTC: Medullary thyroid carcinoma
NETs: Neuroendocrine tumors
NF1: Neurofibromatosis type 1
NF2: Neurofibromatosis type 2
NRs: Nephrogenic rests
OPG: Optic pathway glioma
PHTS: PTEN hamartoma tumor syndrome
RCC: Renal cell carcinoma
US: Ultrasound
VHL: Von Hippel-Lindau
WI: Weighted image
WT: Wilms tumor
